# Increasing the amylose content of durum wheat through silencing of the *SBEIIa *genes

**DOI:** 10.1186/1471-2229-10-144

**Published:** 2010-07-14

**Authors:** Francesco Sestili, Michela Janni, Angela Doherty, Ermelinda Botticella, Renato D'Ovidio, Stefania Masci, Huw D Jones, Domenico Lafiandra

**Affiliations:** 1 University of Tuscia, Department of Agrobiology & Agrochemistry, Viterbo, Italy; 2 Rothamsted Research, Department of Plant Science, Harpenden, UK

## Abstract

**Background:**

High amylose starch has attracted particular interest because of its correlation with the amount of Resistant Starch (RS) in food. RS plays a role similar to fibre with beneficial effects for human health, providing protection from several diseases such as colon cancer, diabetes, obesity, osteoporosis and cardiovascular diseases. Amylose content can be modified by a targeted manipulation of the starch biosynthetic pathway. In particular, the inactivation of the enzymes involved in amylopectin synthesis can lead to the increase of amylose content. In this work, genes encoding starch branching enzymes of class II (SBEIIa) were silenced using the RNA interference (RNAi) technique in two cultivars of durum wheat, using two different methods of transformation (biolistic and Agrobacterium). Expression of RNAi transcripts was targeted to the seed endosperm using a tissue-specific promoter.

**Results:**

Amylose content was markedly increased in the durum wheat transgenic lines exhibiting *SBEIIa *gene silencing. Moreover the starch granules in these lines were deformed, possessing an irregular and deflated shape and being smaller than those present in the untransformed controls. Two novel granule bound proteins, identified by SDS-PAGE in SBEIIa RNAi lines, were investigated by mass spectrometry and shown to have strong homologies to the waxy proteins. RVA analysis showed new pasting properties associated with high amylose lines in comparison with untransformed controls. Finally, pleiotropic effects on other starch genes were found by semi-quantitative and Real-Time reverse transcription-polymerase chain reaction (RT-PCR).

**Conclusion:**

We have found that the silencing of *SBEIIa *genes in durum wheat causes obvious alterations in granule morphology and starch composition, leading to high amylose wheat. Results obtained with two different methods of transformation and in two durum wheat cultivars were comparable.

## Background

Cereal grains contain a good balance of proteins, fats, carbohydrate, vitamins and minerals required for human growth and health. Unlike other cereals, wheat is rarely consumed in an unprocessed form but prepared into a wide range of end products. Common wheat (*Triticum aestivum *L.) is used in the preparation of bread, noodles, biscuits, and cakes. Durum wheat (*T. turgidum *L. var. *durum*) is used mainly for pasta production but also in an array of other regional foods in Italy, North Africa and West Asia (bread, cous cous, burghoul etc). The processing and end-use quality of wheat-based products depends on different factors such as protein content and composition, grain hardness and starch composition. Starch, the most important polysaccharide in human diet and is the major component of the wheat kernel, representing more than 70% of its dry weight. As well as its importance in the food industry, starch is also used as a raw material for the production of non-food products in the paper, plastic, adhesive, textile, medical and pharmaceutical industries [1].

Reserve starch is accumulated in the amyloplast organelles and is composed of two different glucosidic polymers, amylose and amylopectin. The main differences between these polymers are the degree of polymerization and the number of side branches. Amylose is a linear chain of D-glucose molecules with a low degree of polymerization (< 10^4 ^units), whereas amylopectin shows a higher degree of polymerization (10^5^-10^6 ^units) and which has important implications for function. Amylopectin is the major constituent of starch in wheat endosperm and comprises about 70-80%; with amylose constituting the remaining 20-30%. Amylose and amylopectin are synthesized by two different pathways having a common substrate (ADP-glucose). A granule bound starch synthase (GBSSI) is involved in amylose synthesis, whereas amylopectin is produced by the concerted action of starch synthases (SSI, SSII, SSIII), starch branching enzymes (SBEI, SBEIIa and SBEIIb) and starch debranching enzymes of isoamylase- and limit dextrinase-type (ISA and LD) [2,3].

SBEs are transglycosylase enzymes that catalyze the formation of α-1,6 linkages within the polymer by cleaving an internal alpha-1,4 linkage. In monocots, three starch branching isoforms are found: SBEI, SBEIIa and SBEIIb. In maize, rice and pea, suppression of SBEIIb leads to amylose-extender (*ae*) phenotype, with a very high amylose content (>50%) [4-6], in contrast suppression of SBEI or SBEIIa has no effect on the amount of amylose [7-9]. In wheat *SBEIIa *and *SBEIIb *genes have been characterized and found to be located on the long arm of the homoeologous group 2 chromosomes [10-12]. Regina *et al*. [12] demonstrated that wheat *SBEIIa *gene is syntenic to the corresponding gene in other cereals, in contrast the *SBEIIb *gene is not in a syntenic position. In wheat, SBEIIa is the predominant isoform present in the soluble phase of the endosperm [12], whereas in maize and rice endosperm SBEIIb is the predominant isoform involved in amylopectin biosynthesis [13,14].

The role of SBEIIa and SBEIIb isoforms in bread wheat endosperm has been investigated by RNA interference technology [15]. In contrast to other cereals, the silencing of *SBEIIb *genes has no effect on amylose content and starch granule shape; whereas silencing of *SBEIIa *genes results in a strong increase in amylose content (>70%) and granule deformation.

There is increasing interest in the manipulation of starch composition in wheat due to the recognition of its important role in food and non food applications and its uses in industry. In addition, the research is also focusing on the production of high amylose starch flours because derived foods have an increased amount of resistant starch which has been shown to have beneficial effects on human health. Resistant starch refers to the portion of starch that resists digestion in the stomach and small intestine and it can act as a substrate for microbial fermentation in the large intestine, the end-products being hydrogen, carbon dioxide, methane and short chain fatty acids (SCFA) [16,17]. The nutritionists believe that the resistant starch has a role similar to dietary fibre inside the intestine, protecting against diseases as colon cancer, type II diabetes, obesity and osteoporosis [17-21].

A recent study demonstrated that high-amylose flours might be used to substitute for up to 50% normal wheat flour to make bread with both an acceptable bread quality and a significantly higher amount of resistant starch [22].

Furthermore, pasta produced with semolina containing a higher amylose content shows good cooking resistance and firmness, satisfying consumer preferences [23].

In the present study, a transgenic approach was used to increase the amylose content in durum wheat seeds. In particular, the effects of *SBEIIa *silencing were investigated in terms of amylose content, transcript accumulation and protein profile of the enzymes involved in starch biosynthesis.

## Results

### Biolistic transformation and selection of transgenic plants

In total, 1954 immature embryos obtained from the *Triticum durum *cv. Svevo were co-transformed using pRDPT + *SBEIIa*(RNAi) and *bar *selectable marker plasmids. Forty eight T_0 _independent transgenic lines resistant to bialaphos and containing the RNAi cassette were obtained, with a co-transformation efficiency of 2.5%. The presence of the transgene was verified by polymerase chain reaction (PCR) analysis of genomic DNA from regenerated plants, by using two primer pairs, one specific for the promoter of the construct (PromDx5Fw/PromDx5R) and another for the *bar *gene (BarFw/BarR), to produce amplicons of 473 bp and 405 bp, respectively (data not shown). No significant differences in morphology and growth were observed between transgenic lines and either the corresponding "null" genotype that had lost the transgene by segregation (null segregant), or untransformed plants.

### Agrobacterium-mediated transformation and selection of transgenic plants

In all, 1.759 immature embryos of the *Triticum durum *cv. Ofanto were transformed using pGUB-G + *SBEIIa*(RNAi) containing also the *bar *gene cassette in the T-DNA. Thirteen T_0 _transgenic lines were obtained with a transformation efficiency of 0.74%. The presence of the transgene was verified as described above. As with transgenic plants generated by biolistic transformation, no morphological differences were observed among the different lines or between transgenics and controls.

### Molecular characterization of transgenic plants

The absence of *SBEIIa *transcript was investigated by semiquantitive RT-PCR and Real Time RT-PCR on total RNA, extracted from T_2 _immature kernels (18 DPA), using a primer pair specific for *SBEIIa *genes (*SBEIIa*Fw/*SBEIIa*R). We focused on six transgenic lines, three derived from the biolistic method and three from Agrobacterium-mediated transformation. *GADPH *(glyceraldehyde 3-phosphate dehydrogenase) was used as housekeeping gene.

**Figure 1:**
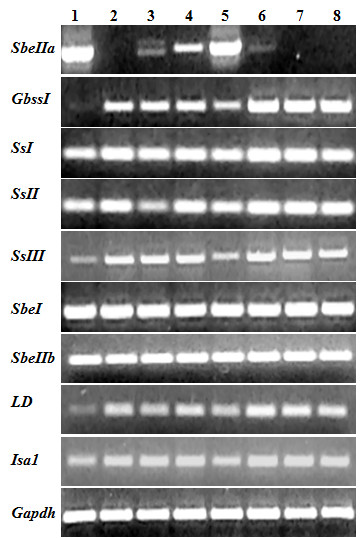
**Semiquantitative RT-PCR of transcripts encoding starch biosynthetic enzymes**. 1.5% agarose gel of specific amplicons of starch genes in total RNA of wheat endosperm collected at 18 dpa. 1) cv Svevo, and the corresponding SBEIIa RNAi lines: 2) MJ16-112; 3) MJ16-114; 4) MJ16-119. 5) cv Ofanto, and the corresponding SBEIIa RNAi lines: 6) A428, 7) A431; 8) A432. Gadph was used as housekeeping gene. SbeIIa, Starch branching enzyme IIa; GbssI, Granule bound starch synthase I; SsI, Starch synthase I; SsII, Starch synthase II; SsIII, Starch synthase III; SbeI, Starch branching enzyme I; SbeIIb, Starch branching enzyme IIb; LD, Limit dextrinase; Isa1, Isoamylase 1; Gadhp, glyceraldehyde-3-phosphate dehydrogenase.

**Figure 2:**
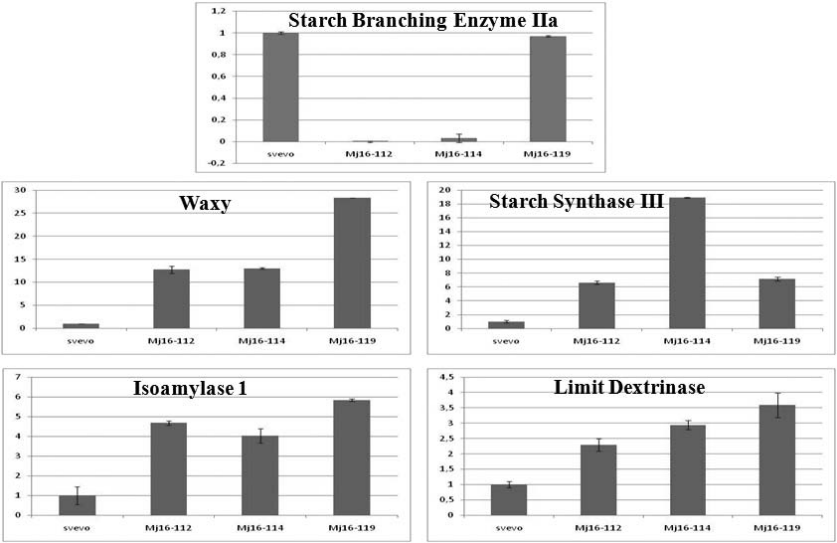
**Real Time quantitative RT-PCR analysis of SBEIIa, GBSSI, SSIII, LD and ISA1 genes.** Transcript levels were evaluated on cDNA obtained from total RNA of immature seeds (18 dpa). The analysis were performed on cv Svevo and the corresponding SBEIIa RNAi lines MJ16-112, MJ16-114 and MJ16-119.

A complete silencing of *SBEIIa *genes was observed in four lines and partial silencing in two lines (Figure 1). Both the partially silenced lines were obtained by biolistic bombardment while the three lines obtained through Agrobacterium-mediated transformation were completely silenced. In one plant (MJ16-114) the level of *SBEIIa *transcript was 30-fold less than in the control line, while in line MJ16-119 the expression of the target gene appeared to be unaffected at 18 DPA (Figure 2).

**Figure 3:**
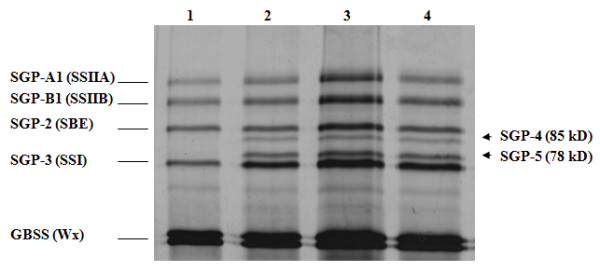
**SDS-PAGE of starch granule proteins.** Starch proteins were extracted from half seed as reported in Methods. 1) cv Svevo, and the corresponding SBEIIa RNAi lines: 2) MJ16-112; 3) MJ16-114 and 4) MJ16-119. The arrows indicate the two novel proteins, SGP-4 and SPG-5, of about 85 and 78 KDa.

Starch granule-bound proteins, extracted from transgenic seeds, were separated on SDS-PAGE gel and compared with those present in the untransformed cultivars. Unexpectedly, two new starch granule-bound proteins were identified in the transformed lines (Figure 3), showing a molecular weight between 78 and 85 KDa. Moreover, the intensity of SGP-3, and to a lesser extent SGP-2, SGP-1 and waxy was higher in the transgenic lines.

The two novel SGPs (SGP-4 and SGP-5) were investigated by mass spectrometry and shown to be waxy-like proteins (Table 1). SGP-4 and SGP-5 were similar to durum wheat genbank accession BAA88512 with a coverage of 41% (247/604 aa) and 36% (215/604 aa), respectively. In both cases the probability that the two proteins corresponded to accession BAA88512 was 100%.

**Table 1:** Peptides identified by mass-mass spectrometry on SGP-4 and SGP-5 proteins.

Pepti de N°	Actual mass (MH+)	Theor mass (MH+)	From-To	Sequence
**SGP-4**				
1	814.29	815.46	304-311	K>AGILQADK<V
2	1443.8	1443.80	39-52	A>ALVMRTIGASAAPK<Q
3	1348.47	1349.66	125-136	K>DAWDTSVVSEIK<V
4	1395.82	1396.74	380-392	K>EALQAEVGLPVDR<K
5	1564.04	1564.76	479-491	R>FEPCGLIQLQGMR<Y
6	2231.62	2232.09	458-478	R>FNAPLAHQMMAGADVLAVTSR<F
7	1685.30	1684.80	266-279	R>FSFDDFAQLNLPDR<F
8	1477.29	1476.75	192-204	R>FSLLCQAALEAPR<I
9	1483.91	1482.82	408-421	K>GPDVMIAAIPEILK<E
10	1827.14	1826.82	176-191	K>IYGPDAGTD YEDNQLR<F
11	3494.18	3492.88	403-434	R>LEEQKGPDVMIAAIPEILKEEDVQIVLLGTGK<K
12	1545.07	1544.76	520-533	R>LSVDCNVVEPADVK<K
13	1672.88	1672.86	520-534	R>LSVDCNVVEPADVKK<V
14	1831.94	1831.85	282-297	K>SSFDFIDGYDKPVEGR<K
15	821.07	821.37	513-519	K>TGFHMGR<L
16	1977.70	1977.98	92-112	K>TGGLGDVLGGLPPAMAANGHR<V
17	881.01	881.40	137-143	K>VADEYER<V
18	1997.26	1997.02	312-329	K>VLTVSPYYAEELISGEAR<G
19	815.84	816.53	535-541	K>VVTTLKR<A
**SGP-5**				
1	814.5	815.46	304-311	K>AGILQADK<V
2	1348.95	1349.66	125-136	K>DAWDTSVVS EIK<V
3	1395.62	1396.74	380-392	K>EALQAEVGLPVDR<K
4	2214.29	2213.11	186-204	Y>EDNQLRFSLLCQAALEAPR<I
5	2084.28	2083.96	174-191	K>EKIYGPDAGTD YEDNQLR<F
6	1564.24	1564.76	479-491	R>FEPCGLIQLQGMR<Y
7	2231.58	2232.09	458-478	R>FNAPLAHQMMAGADVLAVTSR<F
8	1476.17	1476.75	192-204	R>FSLLCQAALEAPR<I
9	1123.02	1123.49	330-338	R>GCELDNIMR<L
10	1826.08	1826.82	176-191	K>IYGPDAGTDYEDNQLR<F
11	1099.04	1099.70	393-402	R>KVPLV AFIGR<L
12	1545.25	1544.76	520-533	R>LSVDCNVVEPADVK<K
13	1673.51	1672.86	520-534	R>LSVDCNVVEPADVKK<V
14	1831.15	1831.85	282-297	K>SSFDFIDGYDKPVEGR<K
15	1979.22	1977.98	92-112	K>TGGLGDVLGGLPPAMAANGHR<V
16	1996.44	1997.02	312-329	K>VLTVSPYYAEELISGEAR<G
17	815.4	816.53	535-541	K>VVTTLKR<A
18	1236.44	1237.61	319-329	Y>YAEELISGEAR<G
19	2155.20	2156.00	492-512	R>YGTPCACASTGGLVDTIVEGK<T

### Determination of total starch, amylose and protein content

**Figure 4:**
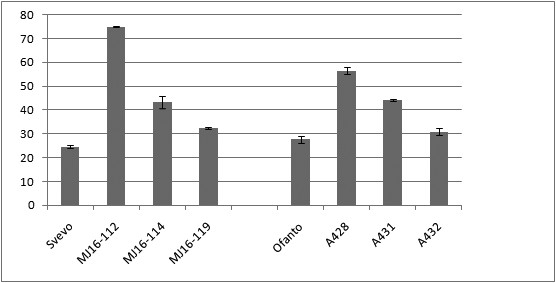
**Amylose content of SBEIIa RNAi lines and untransformed cultivars (cv Svevo and cv Ofanto).** MJ16-112, MJ16-114 and MJ16-119 correspond to transgenic lines obtained from particle bombardment of cv Svevo; A428, A431 and A432 transgenic lines derived from Agrobacterium-mediated transformation of cv Ofanto.

The amylose content was strongly increased in SBEIIa RNAi transgenic lines in comparison to untransformed controls of Svevo and Ofanto. Amylose values ranged from 30.8% up to 75% (Figure 4; Table 2). Although the amylose percentage was more than 70% in endosperm from the MJ16-112 line, the plant morphology, shape and yield of seeds (Table 2) was not significantly different from non-transformed controls. A reduction in the endosperm starch content of transgenic lines obtained by biolistic transformation was observed (Table 2). The protein content of the same lines ranged between 16.16% and 18.95% and were similar to untransformed control (18.36%) (Table 2).

**Table 2:** Seed weight, total starch, amylose and protein content of RNAi transgenic lines and control plants.

Lines	100 grain weight	Starch content %	Amylose content %	Protein content%
Svevo	4.14 ± 0.14	59.80 ± 0.10	24.50 ± 0.70	18.36 ± 0.13
MJ16-112	3.90 ± 0.12	50.98 ± 0.68	75.05 ± 0.36	18.95 ± 0.36
MJ16-114	3.74 ± 0.10	44.91 ± 2.36	43.50 ± 2.52	16.16 ± 0.30
MJ16-119	4.06 ± 0.22	45.17 ± 1.47	32.40 ± 0.39	17.38 ± 0.16
Ofanto	nd	nd	27.69 ± 1.49	nd
A428	nd	nd	56.44 ± 1.43	nd
A431	nd	nd	44.25 ± 0.46	nd
A432	nd	nd	30.87 ± 1.52	nd

Values represent means of three replicates. Standard error is also reported for each value. Nd: not determined.

### Starch granule analysis

**Figure 5:**
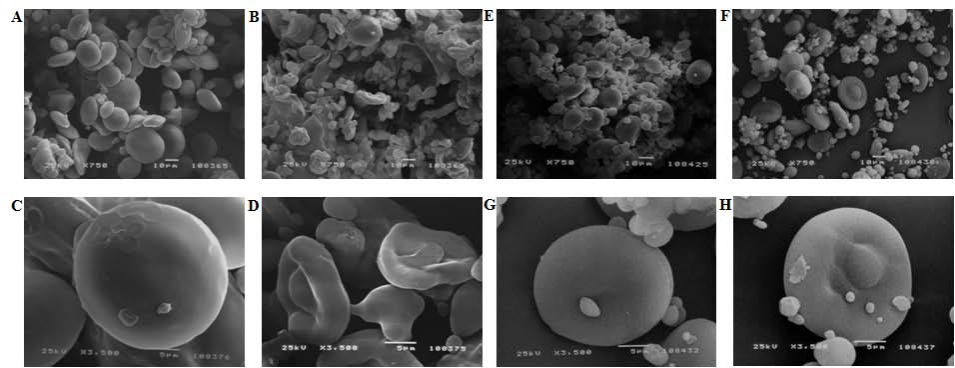
**Scanning electron micrographs of isolated starch granules.** A) and C) nontransformed control wheat cv Svevo; B) and D) SBEIIa RNAi MJ16-112 line; E) and G) nontransformed control wheat cv Ofanto; F) and H) SBEIIa RNAi A432 line.

SEM analysis showed deep alterations of starch granules in MJ16-112 line. The granules with reduced *SBEIIa *expression were deformed with irregular shape, looked deflated and were smaller than wild type (Figure 5). In particular, a high number of A-type granules had a size of 10 μm and B-type granules had lost the spherical shape typical of the controls (Figure 5). This phenotype was observed both in Svevo and Ofanto transgenic lines and is clearly visible in Figure 5. Deformation of starch granules was more evident in line MJ16-112 compared to A432, this could be associated to the different level of amylose content existing between the two lines.

### RVA analysis

**Figure 6:**
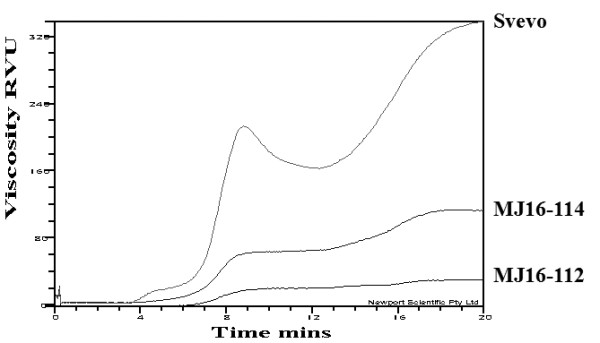
RVA viscograms of control (Svevo) and SBEIIa-RNAi lines (MJ16-112 and MJ16-114) whole wheat flours.

Viscosity properties of whole wheat flours were measured by the Rapid Visco Analyser (RVA) on Svevo and the silenced lines MJ16-112 and MJ16-114. In the case of the high-amylose lines, an overall decrease in viscosity was observed in comparison with the non-transgenic line (cv Svevo). Significant variations were observed for all RVA parameters. Mean trough (TG), breakdown (BD), setback (SB) and final viscosities (FV) of SBEIIa silenced lines were significantly lower than for the control wheat samples (Figure 6; Table 3). There was a clear difference in the retrogradation rates of starches between Svevo and the corresponding transgenic lines, MJ16-112 and MJ16-114. Even though SBEIIa RNAi lines had high amylose content, the setback value was smaller than the control, suggesting that a lower retrogradation of these RNAi lines is due to their lower viscosity value. We observed a negative correlation between amylose percentage and viscosity; in particular the line MJ16-112 had the higher amylose content (75%) and the lower viscosity values. Whereas the line MJ16-114, possessing 43.5% of amylose, had a intermediate RVA profile compared to Svevo and MJ16-112.

**Table 3:** RVA parameters of durum wheat cultivar Svevo and RNAi transgenic lines (MJ16-112 and MJ16-114).

Sample	PV	BD	FV	SB	PT
Svevo	213,66	50,5	338	174,83	8,79
MJ16-112	20,25	2,1	29,1	11	9,93
MJ16-114	64,16	1,58	113,41	50,83	9,99

PV, peak viscosity; BD, breakdown; FV, final viscosity; SB, setback; PT, peak time.

### Real Time RT-PCR on genes involved in starch biosynthesis

To understand if there were pleiotropic effects on the expression of other genes involved in starch biosynthesis, semiquantitative RT-PCR was performed on *GBSSI*, *SSI*, *SSII*, *SSIII*, *SBEI*, *SBEIIb*, *ISA1 *and *LD *genes. The expression levels of many starch biosynthetic genes were affected by the silencing of *SBEIIa *genes. Compared to the control lines, the transcripts of the genes encoding GBSSI, SSIII, LD and ISA1 were increased in transgenic plants, whereas significant changes were not found in *SSI*, *SSII*, *SBEI *and *SBEIIb *genes.

Transcript accumulation of *GBSSI*, *SSIII*, *LD *and *ISA1 *were investigated by quantitative Real-Time RT-PCR. As expected, all analyzed genes were up-regulated in comparison with untransformed plants but their level of up-regulation varied (Figure 2). The expression of *GBSSI *was strongly increased in transgenic seeds with a 12-28 fold increase compared to controls). The expression of *SSIII*, *LD *and *ISA1 *was up-regulated by 6-18, 1,5-3,5 and 4-6 fold respectively in Svevo transgenic plants compared to controls (Figure 2).

## Discussion

Different strategies can be used to modify amylose content in wheat starch. In the last twenty years the identification of *null *mutations in starch biosynthetic genes and the crossing of these materials to combine functional mutations have permitted the production of lines with low and high amylose content [24-26]. Unfortunately this strategy is not easy to apply for all starch biosynthetic proteins, due to the difficulty of obtaining the relevant mutants in polyploid species such as wheat.

Both transgenic and non-transgenic tools are available, and the latter, along with novel technologies, offers the possibility to manipulate starch composition and develop wheat varieties with new functionalities. Recently a reverse genetic strategy called TILLING (Target Induced Local Lesions in Genomes) has permitted the identification of induced mutations for genes involved in amylose [27] and amylopectin [28] biosynthesis. The TILLING strategy is an efficient, non-transgenic method to identify mutants in genes of interest, but the effect of silencing is often masked by the presence of other functional alleles in polyploid species. To obtain a *null *phenotype, it is usually necessary to generate double or triple mutants by crossing single *null *genotypes [29].

Recent studies have demonstrated that transgenic technology is a powerful research tool to modify amylose/amylopectin ratio. Starch composition can be manipulated through genetic engineering, either by overexpression or repression of starch biosynthetic genes. RNAi technology is a specific and powerful mean to silence a target gene. This technology is particularly useful in polyploid plants such as wheat because it increases the possibility to obtain transgenic plants with a novel phenotype.

In this study, an RNAi strategy has been followed to silence *SBEIIa *genes in order to increase the amylose content in two durum wheat cultivars, Svevo and Ofanto. Although two different methods have been used for genetic transformation (biolistic for cv Svevo and Agrobacterium for cv Ofanto), the silencing of *SBEIIa *genes produced similar effects. The Agrobacterium- or the biolistic-mediated genetic transformation showed various levels of silencing between independent transgenic wheat lines. The abundance of mRNAs of *SBEIIa *genes varied in the transgenic lines from nearly undetectable to wild type level confirming that the RNAi construct, designed to reduce the expression of *SBEIIa *genes, was effective in inducing gene silencing. The variation in the levels of gene silencing observed here is in agreement with previously reported results in wheat [30,31] and other species, such for example tobacco [32], maize [33] and Arabidopsis [34].

The silencing of the *SBEIIa *gene caused marked modifications on amylose content, granule morphology and starch composition. An increase in amylose content was found in all transgenic lines but its percentage varied between lines, presumably due to differences in copy number and/or positional effects. The line MJ16-112 showed the exceptionally high value of 75% amylose content, while in the other lines it varied between 31-56%. A variable level of amylose content between RNAi lines was also found in sweetpotato [35] and potato [36].

No significant difference was observed in grain yield between transformed and control lines, although RNAi lines showed a lower total starch content. The reduction of total starch in RNAi lines could be due to the difficulty to estimate starch content in high amylose wheats. McClearly et al. [37] used different approaches to determine total starch content and found that in high amylose maize the starch content resulted always underestimated.

Morphological analysis of starch granules showed a marked difference between *SBEIIa*-silenced lines and control plants. Type-A granules appeared smaller and deflated, whereas type-B granules lost their normal spherical shape and became extended. Similar results were obtained by Regina *et al. *[15] who suppressed the activity of SBEIIa through RNAi technology in bread wheat.

RVA analysis, carried out on whole wheat flour, highlighted large alterations in chemical-physical properties of transgenic starch. RVA parameters, as mean trough, breakdown, setback and final viscosities of *SBEIIa*-silenced lines, were significantly lower than normal wheats. These results are consistent with those obtained on high amylose flour of wheat [38], corn [39] and barley [40]. The decreased swelling and viscosity affects chemical-physical pasting properties of semolina, with subsequent changes in functional characteristics of derived food products. Although high amylose flour resulted in a smaller bread loaf volume [41], it has a positive impact in the pasta industry, reducing starch loss during the cooking and increasing pasta firmness. In addition, high amylose foods might have beneficial effects on human diet because of an increased amount of resistant starch, which has a role similar to dietary fibre inside the intestine, protecting against diseases as colon cancer, type II diabetes, obesity and osteoporosis [17,18].

SDS-PAGE analysis of starch granule-bound proteins allowed the identification of two newly accumulated proteins localized between the SGP-2 and SGP-3 in the transgenic lines. These novel proteins are present in all transgenic plants, suggesting that they are a consistent result of *SBEIIa *silencing rather than an unintended effect of random transgene insertions. The novel proteins show a high similarity to the waxy isoforms. Since the molecular mass of the novel proteins is larger (85 and 78 KDa) than that of waxy proteins (58-60 KDa), one possibility is that the novel proteins are encoded by genes that are poorly or not expressed in wild type cultivar. An alternative explanation is that a mechanism of post-translation modification has somehow been triggered in these transgenic lines.

The staining intensity of the other starch granule proteins (SGP-140, SGP-145, SGP-1, SGP-3 and waxy) on SDS-PAGE is strongly increased in the silenced lines compared to the control. Real-Time RT-PCR analyses on genes involved in starch biosynthesis have confirmed possible interaction and feedback mechanisms between these genes. In particular, the absence of SBEIIa isoforms leads to an up-regulation of *Wx*, *SSIII*, *LD *and *ISA1 *genes.

Pleiotropic effects associated with up-regulation or suppression of specific genes present in the starch biosynthetic pathway have been reported previously. For example, Kosar-Hashemi *et al. *[42] observed a reduction of granule-bound branching enzymes and starch synthases in the starch granule of the *SSII *mutants and suggested that the lack of *SSII *would lead to the absence of the complex on starch granule. Similarly, the rice *sugary-1 *(null for isoamylase) and the maize *dull-1 *(not expressing *SSIII*) mutants had drastic changes in accumulation of pullulanase and SBEIIb isoforms, respectively [43,44]. In a recent work, the down-regulation of a pullulanase-type DBE inhibitor produced pleiotropic effects on activities of soluble and granule-bound starch synthase, that were significantly reduced [45]. Tetlow *et al. *[46] have discovered the formation of a heterocomplex comprising SBEI, SBEIIb, and starch phosphorylase in wheat. More recently, Tetlow *et al*. [47] have identified trimeric assemblages of SSI with SSIIa and either SBEIIa or SBEIIb. Hennen-Bierwagen *et al*. [48,49] have demonstrated that SSs, SBEs and DBEs associate physically with each other in multisubunit complexes and the lack of one subunit can cause variation on the other isoforms. In maize the *ae*^- ^mutation, in the gene coding for SBEIIb, was associated with the detection of four novel proteins not observed in wild-type granules. LC-MS/MS analysis showed that the four bands corresponded to SBEI, SBEIIa, SSIII and starch phosphorylase [50]. Grimaud *et al. *[50] suggested that structural changes in amylopectin, due to the loss of SBEIIb, could offer a more efficient glucan substrate for binding of SBEI, SBEIIa, starch phosphorylase and SSIII. The pleiotropic effects observed in our *SBEIIa*-silenced lines represent additional evidence of a strict correlation between all starch enzymes and that the lack of one subunit can cause variation in the other isoforms.

## Conclusions

The RNAi-mediated down-regulation of genes coding the SBEIIa isoforms produced remarkable changes in starch chemical-physical properties of durum wheat. No significant differences were identified in grain weight between transgenic plants and wild type controls. As well as a considerable increase in the amylose content, the silenced *SBEIIa *wheat lines also possessed altered starch granule structure and up-regulated transcriptional profiles of GBSSI, SSIII, LD and ISA1. The use of two different transformation methods, biolistic and Agrobacterium-mediated, and two different durum wheat cultivars, highlights that the effect of *SBEIIa *silencing on starch metabolism is genotype and protocol independent because similar effects have been detected in all the RNAi plants obtained.

The variation in amylose content and the different starch physical-chemical properties make these plant materials particularly valuable in a vast range of applications in both food and non food industries.

## Methods

### Plant material

Durum wheat plants (cv Svevo and cv Ofanto) were vernalized at 4°C for one month and grown in a growth chamber at 20/18°C day/night temperature under a 16/8 h photoperiod.

### Vectors for biolistic and Agrobacterium-mediated transformation

The first three exons, of the wheat *SBEIIa *gene were amplified by RT-PCR on total RNA extracted from immature seeds (18 dpa), while the third intron of the wheat *SBEIIa *gene was amplified from genomic DNA of Svevo. The primers used were: *SalIXbaI*SBEII(cDNA)FW 5'-gtcgactctagaggcgggttgagtgagatctg; *KpnIXhoI*SBEII(cDNA)R 5'-ggtaccctcgagtcggtagtcaagatggctcc; *SalIKpnI*SBEII(INT)FW 5'-gtcgacggtaccgcagaaaatatacgagattg; *XbaIXhoI*SBEII(INT)R 5'-acctctagactcgagccaccttcatgttggtcaatag.

cDNA synthesis was performed by using the QuantiTect Reverse Transcrition Kit (Qiagen) following the manufacturer instructions.

PCR reactions were carried out in 50 μl final volume using 50-100 ng of genomic DNA or 1 μl of cDNA, 2.5 units of FastStart High Fidelity PCR system (Roche Diagnostics), 1× Taq PCR buffer, 50 ng of each of the two primers and 100 μM of each deoxyribonucleotide. PCR conditions were: 1 cycle at 95°C for 2 min, 35 cycles at 95°C for 30 s, 60°C for 1 min, 72°C for 1 min, and a final step at 72°C for 5 min.

The intron was ligated in the vector pRDPT containing the promoter and terminator of the Dx5 high molecular weight glutenin subunit gene [51]. Then taking advantage of restriction sites introduced on the primers, the sense and antisense sequences of the target region were inserted in the plasmid pRDPT separated each one by the intron. This construct was used for biolistic transformation.

The entire cassette (promoter-sense-intron-antisense-terminator) was extracted from pRDPT vector by digestion with *SmaI *and *HpaI *restriction enzymes and cloned into the *SmaI *site of the vector pG-UB carrying the *bar *gene that confers resistance to the bialaphos herbicide under the ubiquitin promoter. This construct was used for Agrobacterium-mediated transformation.

### Biolistic and Agrobacterium transformation of durum wheat embryos

For the transformation experiments, the plasmid pAHC20 [52], carrying the *bar *gene, was co-bombarded with pRDPT + *SBEIIa*(RNAi) in a 1:3 molar ratio. Constructs were introduced into immature embryos of *T. durum *cv. Svevo excised from seeds in Zadoks stage 72, using a Model PDS-1000/He Biolistic particle delivery system (Bio-Rad, Hercules, CA, U.S.A.) and the protocols described in Okubara and associates [53]. The protocol of Wu *et al. *[54] was used for transformation of durum wheat Ofanto by Agrobacterium. The presence of the construct in bialaphos-resistant plants and their progeny was verified by PCR on total DNA obtained from leaf sections of mature plants [55] with primer pairs specific for the promoter Dx5 and for the *bar *gene (PromDx5Fw catgcaggctaccttccac, PromDx5R cggtggactatcagtgaattg, BarFw catcgagacaagcacggtca and BarR gaaacccacgtcatgccagt). T2 non segregating progeny were used for molecular and biochemical analyses (additional file 1).

PCR reactions were carried out in 50 μl final volume using 50-100 ng of genomic DNA, 1× Red Taq ReadyMix PCR REACTION MIX (1.5 U Taq DNA Polymerase, 10 mM Tris-HCl, 50 mM KCl, 1.5 mM MgCl_2, _0.001% gelatine, 0.2 mM dNTPs) and 0.5 μM of each of the two primers.

Amplification conditions included an initial denaturation step at 94°C for 3 min, followed by 35 cycles at 94°C for 1 min, 60°C for 1 min and 72°C for 1 min, followed by a final incubation at 72°C for 5 min.

### Semiquantitative Reverse transcriptase-polymerase chain reaction

Total RNA was extracted from immature seeds (18 DPA) as reported in Laudencia-Chingcuanco *et al*. [56] with some modifications. The starting material was 0.1 g and all volumes of buffers and solutions were diluted 1 to 10. For reverse transcriptase-mediated PCR studies, cDNA was synthesised from 1 μg of total RNA using an oligo(dT) primer and Superscript Reverse Transcriptase III (Invitrogen). One of twentieth volume of each cDNA was used as a template for PCR amplification. PCR reactions were carried out in 30 μl final volume using 1 units of Ex-Taq (Takara), 1× buffer, 0,2 mM of each dNTPs, 0,5 μM of each primer. Amplification conditions included an initial denaturation step at 98°C, followed by 35 cycles at 98°C for 10 sec., 58°C for 1 min. and 72°C for 1 min, followed by a final extension at 72°C for 5 min. The following gene-specific primers were designed for GAPDH (GAPDHFw 5'-caacgctagctgcaccactaact and GAPDHR 5'-gactcctccttgatagcagcctt) [57], GBSSI (Wx-B1Fw 5'-cgaagcaacaaagccggaaag and Wx-B1R 5'-tcaccctctcgtactcgtccg), SSI (SSIFw 5'-agggtacagggtgggcgttct and SSIR 5'-gtagggttggtccacgaagg), SSII (SSIIFw 5'-ttcgaccccttcaaccactc and SSIIR 5'-acgtcctcgtagagcttggc), SSIII (5'-ggcgttggatgtgtatatgg and 5'-ggtgatgattccgacaatagg) [58], SBEI (SBEIFw 5'-aaacaaacttcaacaaccgcc and SBEIR 5'-acctcctgtagacgcctttt), SBEIIa (*SBEIIa*Fw 5'-tgacgaatcttggaaaatgg and *SBEIIa*R 5'-ggcggcatttatcataactattg), SBEIIb (SBEIIbFw 5'-gtagatgcggtcgtttacttga and SBEIIbR 5'-ccagccaccttctgtttgtt), LD (LDFw 5'-tatgagtggaacagggattggt and LDR 5'-atttggtcagcgtaagtagcg) and ISA1 (ISA1Fw 5'-aagatgaaagacagggcgagat and ISA1R 5'-aatactaggatgaccgatgagtagct).

### Real-Time RT-PCR (qRT-PCR)

One microlitre of the cDNA above described was used for real-time PCR in a 20 μL volume. For each sample three technical replicates were used for PCR amplification. The PCR reaction consisted of 10 μL of iQ™ SYBR Green Supermix 2X (BIO-RAD), which contained buffer, dNTPs and SYBR Green I. Concentrations of the forward and reverse oligodeoxynucleotide primers in the reaction were 500 nM for all the genes of interest.

qRT-PCR experiments were performed using the *iCycler iQ *(Bio-Rad Laboratories, Hercules, CA1, USA). Amplification conditions were as follows: initial 95°C for 15 min and 40 cycles of 95°C for 30 s, 60°C for 1 min and 72°C for 1 min each.

Relative expression analysis was determined by using the 2^-ΔΔCT ^method [59]; Applied Biosystems User Bulletin No. 2-P/N 4303859). Calculation and statistical analyses were performed by Gene Expression Macro™ Version 1.1 (Bio-Rad Laboratories, Hercules, CA, USA). The efficiencies of target and housekeeping genes were determined by qRT-PCR on serial dilutions of RNA template over a 100-fold range [59], with similar results (data not shown). Amplified products were checked by gel electrophoresis and sequencing to verify primer specificity. Relative expression of each gene is reported as the number of fold increase of the transcript level at each time point, compared to the lowest transcript level.

As for semiquantitative RT-PCR, *GAPDH *was used as housekeeping gene.

### Estimation of Total starch, Amylose and protein content

Total starch content of grounded kernels was determined by Megazymes Total Starch Assay Kit (AA/AMG, Megazyme Pty Ltd., Wicklow, Ireland).

Amylose content was estimated by iodometric assay as reported in Chrastil [60] by using starch extracted from mature wheat kernels according to procedure described by Zhao and Sharp [61].

Protein content was determined by the Dumas combustion method, using a Leco FP 428 analyzer (Leco Co., St. Joseph, MI) calibrated with an EDTA standard.

### Protein extraction and analysis

The preparation of starch granules from half seeds and the separation of starch granule bound proteins by SDS-PAGE followed the method reported by Zhao and Sharp [61] with some modifications, as in Mohammadkhani *et al*. [62]. Protein bands were visualized by silver staining.

### RVA

Starch paste viscosity was measured by the rapid visco analyzer (RVA series 4, Newport Scientific, Sidney, Australia). Whole durum wheat flour (3,87 g) was mixed with 25,13 mL of 0,2% silver nitrate in a RVA canister. The starch suspension was, constantly, stirred at 160 rpm and heated from 50 to 95°C, held at 95°C for 4 min and then cooled to 50°C for 4 min. The following variables were recorded in duplicate: peak viscosity (PV), breakdown (BK), trough (TG), final paste viscosity (FV), peak time (Pt), pasting temperature (PT), and setback (SB). Viscosity was expressed in Rapid Visco Unit (RVU).

### Mass spectrometry

Starch was isolated from 5 seeds as described in Zhao and Sharp [61] with same modification. Starch was resuspended in 1000 μl extraction buffer (Tris 0,5 M; SDS 10%, glycerol 50%, sucrose and DTT 1%) and boiled for 15 min. Gelled starch solution was then cooled at -20°C for 1 hour and centrifuged at 13000 rpm for 10 min. Four volumes of acetone were added to the supernatant that was stored at -20°C overnight. The samples was centrifuged at 4°C at 8500 rpm for 40 min; pellet was washed with 2,5 ml of aceton for three times, and finally air-dried. Precipitated proteins were resuspended in 35 μl extraction buffer and loaded on SDS-PAGE (T = 10; C = 1,28), running at 200 V for 1 h and 35 minute. Protein bands were visualized by colloidal coomassie CBBG-250 [63], rinsed out with mill-Q water filter, and captured with sterile scalpel. All steps were conducted in sterile atmosphere, making attention to keratin contaminations. The captured bands were placed in a eppendorf and submitted by Mass spectrometry analysis (University of California, Davis, CA, U.S.A). Scaffold (version Scaffold-2.02.01) was used to analyze Tandem MS/MS based peptides and to identify the proteins. Peptide identifications were accepted if they could be established at greater than 95.0% probability as specified by the Peptide Prophet algorithm [64]. Protein identifications were accepted if they could be established at greater than 99.0% probability and contained at least two identified peptides. Protein probabilities were assigned by the Protein Prophet algorithm [65].

The proteins were prepared for MS analysis using standard reduction, alkylation, and tryptic digest procedures [66]. Peptides were dried down, then resolubilized in 2% acetonitrile/0.1% trifluoroacetic acid for LC-MS/MS analysis.

Digested peptides were analyzed by LC-MS/MS on a LTQ-FT with Waters nano Acuity UPLC. Peptides were separated with a 90 min gradient using a Waters 100 um × 100 mm 1.7 um BEH130 C18 reversed phase column at 2 μl/min. The LTQ-FT was operated with a top 4 method, where the first scan was acquired in the FT at 100,000 resolution and the top 4 ions in each survey scan were selected for low energy CID in the LTQ. Solvent A composition was 0.1% formic acid, and solvent B 100% ACN. Peptides were separated at 2 μl/min on the reversed-phase column with this gradient: 0 to 7% Solvent B in 5 minutes, 7-35% in 65 minutes, 35-70% in 5.2 minutes, held at 70% for 0.8 minutes, 70-1% in 0.2 minutes, with 13.8 minutes to equilibrate between runs.

X!Tandem was used to search the MS/MS spectra against both the provided database. A parent mass error of up to 10 ppm was allowed for the searches, and a product mass error of 0.4 Daltons. Complete modification of carbamidomethyl was used, as well as the potential modification of methionine oxidation.

### Microscopic Analyses of Starch Granules

Starch granules were extracted from seeds as reported in Zhao and Sharp [61] with some modifications, as in Mohammadkhani *et al*. [62]. Samples were critical-point-dried in a Balzer's apparatus equipped with a liquid CO_2 _inlet and metal-shadowed in a gold sputtering unit equipped with an argon inlet. Specimens were examined in a Jeol JSM 5200 scanning electron microscope.

## Authors' contributions

FS carried out part of the transformation work, the molecular analyses and drafted the paper with DL and RDO; MJ carried out the biolistic transformation; AD collaborated in the Agrobacterium transformation; EB carried out the biochemical analyses; RDO participated to the biolistic transformation; SM has contributed to the proteomic part of the work; HDJ has collaborated with the Agrobacterium transformation and edited the manuscript; DL conceived and coordinated the work. All authors read and approved the final manuscript.

## Supplementary Material

Additional file 1 **Segregation data of RNAi transgenic lines**. Biochemical analyses have been performed on PCR positive seeds of T_2 _plants. Click here for file
